# Molecular and serological evidence of flea-associated typhus group and spotted fever group rickettsial infections in Madagascar

**DOI:** 10.1186/s13071-017-2061-4

**Published:** 2017-03-04

**Authors:** Rado J. L. Rakotonanahary, Alan Harrison, Alice N. Maina, Ju Jiang, Allen L. Richards, Minoarisoa Rajerison, Sandra Telfer

**Affiliations:** 1 Plague Unit, Institut Pasteur de Madagascar, Antananarivo, Madagascar; 2 Ecole Doctorale Science de la Vie et de l’Environnement, Université d’Antananarivo, Antananarivo, Madagascar; 3 Institute of Biological and Environmental Sciences, University of Aberdeen, Aberdeen, UK; 4 Viral and Rickettsial Diseases Department, Naval Medical Research Center, Silver Spring, MD USA

**Keywords:** *Rickettsia*, Rickettsioses, Fleas, Prevalence, Madagascar, Murine typhus, Flea-borne spotted fever

## Abstract

**Background:**

Rickettsiae are obligate intracellular bacteria responsible for many febrile syndromes around the world, including in sub-Saharan Africa. Vectors of these pathogens include ticks, lice, mites and fleas. In order to assess exposure to flea-associated *Rickettsia* species in Madagascar, human and small mammal samples from an urban and a rural area, and their associated fleas were tested.

**Results:**

Anti-typhus group (TGR)- and anti-spotted fever group rickettsiae (SFGR)-specific IgG were detected in 24 (39%) and 21 (34%) of 62 human serum samples, respectively, using indirect ELISAs, with six individuals seropositive for both. Only two (2%) *Rattus rattus* out of 86 small mammals presented antibodies against TGR. Out of 117 fleas collected from small mammals, *Rickettsia typhi*, a TGR, was detected in 26 *Xenopsylla cheopis* (24%) collected from rodents of an urban area (*n* = 107), while two of these urban *X. cheopis* (2%) were positive for *Rickettsia felis*, a SFGR*. R. felis* DNA was also detected in eight (31%) out of 26 *Pulex irritans* fleas.

**Conclusions:**

The general population in Madagascar are exposed to rickettsiae, and two flea-associated *Rickettsia* pathogens, *R. typhi* and *R. felis*, are present near or in homes. Although our results are from a single district, they demonstrate that rickettsiae should be considered as potential agents of undifferentiated fever in Madagascar.

## Background

Rickettsiae are Gram-negative obligate intracellular bacteria, closely associated with blood-feeding arthropods and subdivided in two groups: typhus group (TGR) and spotted-fever group (SFGR) [1]. They are responsible for many human infections resulting in mild to severe diseases, causing public health problems in many countries around the world. To our knowledge, there have been no confirmed reports of human cases of acute rickettsial infection from Madagascar. However, recent studies have revealed a high prevalence of *Rickettsia africae*, a SFGR, in *Amblyomma* ticks collected from cattle [2] and tortoises [3], as well as evidence of low rates of previous exposure to SFGR in pregnant women [2]. Most SFGR are tick-borne, however *Rickettsia felis*, the etiological agent of flea-borne spotted fever, is commonly associated with fleas [4]. *Rickettsia typhi*, a TGR, is another flea-borne *Rickettsia* and agent of murine typhus [5]. Although there is a lack of information concerning flea-borne *Rickettsia* in Madagascar, human cases of murine typhus and humans seropositive for anti-TGR antibodies have been reported in the neighbouring islands of Reunion and the Comoros archipelago, respectively [6, 7]. The objectives of this study were to assess previous exposure of human populations from Madagascar to both SFGR and TGR, and to determine whether flea-borne rickettsiae circulate in peridomestic communities of small mammals and fleas.

## Methods

**Fig. 1:**
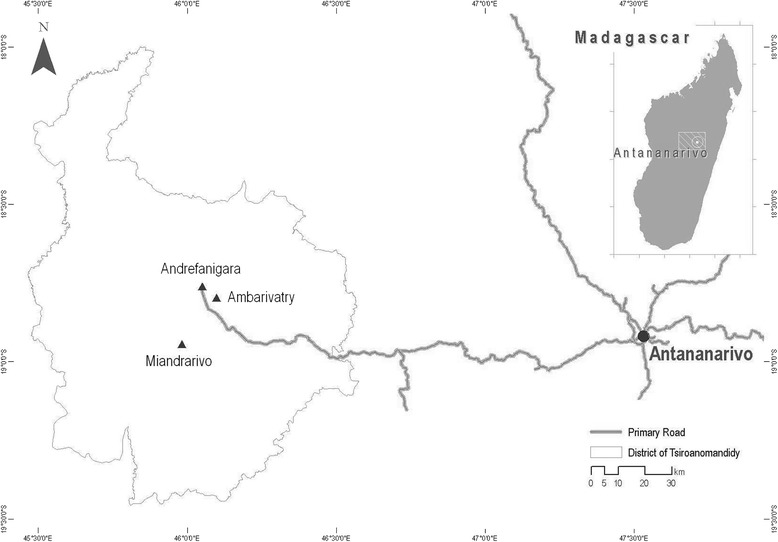
Location of the study sites in Tsiroanomandidy district, central Madagascar. Samples from human subjects, small mammals and small mammal fleas were collected from Andrefanigara and Ambarivatry. Additional flea samples collected using a light trap were collected using a light trap at a second rural village, Miandrarivo

The study was conducted in an urban area, Andrefanigara (18°45′46″S, 46°2′45″E) and a rural area, Ambarivatry (18°47′51″S, 46°5′59″E) within the Tsiroanomandidy district (Fig. 1) in January 2012. In each area, serum samples were collected from 31 healthy human participants. Selection of participants was conducted as follows. In each area, four departure points were randomly selected. For the urban area, the four departure points were located within an 800 m radius of the main health center, whilst for the rural area the four departure points were selected using a schematic diagram of the village overlaid with a grid. At each departure point, a direction of travel was randomly selected and houses then visited sequentially until 7–8 participants had been recruited. A maximum of three persons were recruited from a single household. All participants gave informed consent and were more than 18 years old (National ethical committee authorization 066-MSANP/CE).

To catch peridomestic small mammals, two traps [one wire-mesh trap (BTS) and one Sherman trap] were placed inside 15 and 20 houses in the rural and urban areas, respectively. Where possible a further BTS trap was set in the immediate vicinity of houses (*n* = 7 in the urban area only). Further BTS traps were placed in outside trap-lines close to vegetation, for example adjacent to paths or areas used for growing crops or disposing of waste. Three lines with a total of 50 traps were set in the rural area and three lines with a total of 33 traps were set in the urban area. Thus, a total of 80 traps were set in both areas. Traps were baited with onion, dry fish and carrot and checked each morning for 3 days. Small mammals were identified by morphological characteristics, euthanized and blood samples taken via cardiac puncture. The fur was back-combed using a brush to collect fleas. The study was conducted in accordance with the Institut Pasteur (Paris) animal use guidelines (http://www.pasteur.fr/en/file/2626/download?token=YgOq4QW7) and was approved by the local committee of the Institut Pasteur de Madagascar. Additional flea samples were trapped from 60 houses using light traps [8] during a plague investigation in another rural village within Tsiroanomandidy district, Miandrarivo (18°56′39″S, 45°59′0″E) (Fig. 1) in October 2012. All blood samples were centrifuged and sera stored in liquid nitrogen before laboratory storage at −80 °C. Fleas were stored in 95% ethanol.

To assess previous exposure to *Rickettsia* spp., TGR- and SFGR-specific indirect IgG-ELISAs were conducted using human, rat and shrew sera. ELISAs were performed as described previously [9] with slight modifications. *R. typhi* Wilmington strain and *Rickettsia conorii* Malish strain whole-cell antigen preparations provided by the Naval Medical Research Center (NMRC), Maryland, USA, were used as TGR and SFGR ELISA antigen, respectively. Coating and blocking buffer was PBS-X powder (SIGMA, Saint Louis, MO) dissolved within ultra-pure water (Gibco, Life Technologies, Paisley, UK). Peroxidase labelled anti-human IgG (KPL, Gaithersburg, MD), anti-rat IgG peroxidase-conjugate (Sigma, Saint Louis, Missouri, USA) and protein-A peroxidase (Sigma) were used as secondary antibodies for human, rat and shrew sera, respectively. Secondary antibody dilutions were chosen to optimise the performance of the ELISAs according to the difference in absorbance (optical density, OD) of negative and positive controls. Anti-human secondary antibodies dilutions were 1:4000 for TGR and 1:3000 for SFGR ELISA, while anti-rat conjugate and protein-A conjugate were diluted at 1:2000. The absorbance was read at 405 nm using ELx800 spectrophotometer (Biotek, Winooski, VT) after 15 min of incubation. On each plate, one positive control and three negative controls were included. The positive controls for TGR and SFGR (human and mouse) were provided by NMRC. The human negative controls for TGR and SFGR were pools of Malagasy human sera confirmed negative for TGR and SFGR at the NMRC. The negative rodent controls were obtained from black rats born in captivity at Institut Pasteur de Madagascar. A sample was considered positive when the ratio (net OD/mean three negative controls + 3SD) ≥ 2. Positive samples were subsequently included in a titration ELISA with four-fold dilution of the sera from 1:100 to 1:6400 to determine the individuals’ antibody titer.

Flea species were identified using a dissecting microscope using morphological keys for all fleas described in Madagascar [10]. DNA was individually extracted using an in-house protocol. Briefly, fleas were dried, added to 150 μl of brain-heart infusion broth, macerated with 5 mm steel beads using a Tissue-Lyser II (Qiagen, Hilden, Germany), and then centrifuged at 14,000 rpm for 10 s. The extraction then followed a previously described technique [11], with DNA dissolved in 60 μl of Tris-EDTA (pH 8). To detect DNA contamination, a negative control (water) was included for each 10 fleas.

All fleas collected from small mammals (*n* = 117) and one flea per house in Miandrarivo (*n* = 26) were screened for the presence of *Rickettsia* spp. using the genus-specific real-time PCR assay with RKND03F (5′-GTG AAT GAA AGA TTA CAC TAT TTA T-3′), RKND03R (5′-GTA TCT TAG CAA TCA TTC TAA TAG C-3′) and RKND03P (5′-FAM-CT ATT ATG CTT GCG GCT GTC GGT TC-TAM RA-3′), based on the *gltA* gene [12]. *Rickettsia-*positive fleas were subsequently assessed using two real-time PCR assays targeting the *ompB* genes of *R. typhi* using RT557F (5′-TGG TAT TAC TGC TCA ACA AGC T-3′), RT678R (5′-CAG TAA AGT CTA TTG ATC CTA CAC C-3′) and RT640BP (5′-FAM-CG CGA TCG TTA ATA GCA GCA CCA GCA TTA TCG CG-TAM RA-3′); and *R. felis* with RF1396F (5′-ACC CAG AAC TCG AAC TTT GGT G-3′), RF1524R (5′-CAC ACC CGC AGT ATT ACC GTT-3′) and RF1448BP (5′-FAM-CG CGA CTT ACA GTT CCT GAT ACT AAG GTT CTT ACA GGT CGC G-T AMR A-3′) [13]. Samples with a cycle threshold (Ct) lower than 40 were considered positive. Thus, to be considered positive for TGR or SFGR, a sample had to have a Ct lower than 40 for both the genus-specific and species-specific assays. Each assay was run with negative and positive controls. Negative controls were distilled water and negative controls from DNA extraction. Positive controls were *Rickettsia montanensis* DNA for genus-specific qPCR, and plasmids containing target sequence of *R. typhi* and *R. felis ompB* fragment for species-specific qPCR assays.

To obtain amplicons suitable for sequencing, a subset of samples were chosen to be representative of results from the genus-specific and species-specific qPCR assays. These included *Xenopsylla cheopis* and *Pulex irritans* fleas and a range of Ct values. These samples were included in a semi nested-PCR assay targeting a fragment of *ompB*. The first round PCR was run with primers 120-M59F (5′-CCG CAG GGT TGG TAA CTG C-3′) and 1570R (5′-TCG CCG GTA ATT RTA GCA CT-3′) [14, 15]. In the second round, 120-807R (5′-CCT TTT AGA TTA CCG CCT AA-3′) [14] was paired with 120-M59F which amplify a fragment of approximately 833 bp of *ompB* gene, and 1 μl of first round PCR products were used as template. PCR products were visualized on 1.5% agarose gel, purified with an Isolate PCR and Gel Kit (Bioline, London, UK) and sequenced in both directions using a commercial sequencing service (Eurofins, Ebersberg, Germany). Sequences were aligned and phylogenetic analyses performed using Mega 6.0 [16], analyses included *ompB* sequences from positive fleas and reference sequences from rickettsiae retrieved from GenBank (https://www.ncbi.nlm.nih.gov/genbank/). The phylogenetic trees were constructed using maximum likelihood method and the bootstrap analyses were performed with 1000 replications.

Statistical analyses on serological and qPCR data were conducted using R software [17]. Associations were tested using Fisher exact tests. A *P*-value less than 0.05 was considered significant.

## Results

Fifty-two small mammals were captured from Andrefanigara (44 *Rattus rattus*; 6 *Mus musculus*; 2 *Suncus murinus*) and 41 rodents from Ambarivatry (40 *R. rattus*; 1 *M. musculus*). From urban small mammals, 111 fleas were collected from 22 rodents, consisting of 107 *Xenopsylla cheopis* and 4 *Synopsyllus fonquerniei*. Rats from houses and their immediate vicinity were more often infested with *X. cheopis* (87.5%, *n* = 16) than those from outdoor lines around vegetation (17.9%, *n* = 28; *P* < 0.001, odds ratio = 28.62; 95% CI: 4.60–336.22). Six fleas were obtained from 3 *R. rattus* trapped in Ambarivatry, including 1 *X. cheopis* and 5 *S. fonquerniei*. In Miandrarivo, *Pulex irritans* were found in 26 (43.3%) out of 60 houses.

Overall 39 and 34% of human serum samples tested positive for IgG against TGR and SFGR respectively (Table 1). There was no significant difference in prevalence between urban and rural areas for either TGR (*P* = 0.43) or SFGR (*P* = 0.59). There was also no evidence of an increase in seroprevalence with age for TGR [18–24 years 35% (*n* = 12); 25–34 years 29% (*n* = 14); 35–49 years 58% (*n* = 12); > 49 years 38% (*n* = 16); *P* = 0.49] or SFGR (18–24 years 35%, 25–34 years 36%, 35–49 years 8%, > 49 years 50%; *P* = 0.13). Evidence of a difference in seroprevalence for SFGR between males and females approached significance (males 44% (*n* = 39), females 19% (*n* = 23); *P* = 0.052). No such pattern was observed for TGR (males 33%, females 48% (*n* = 23); *P* = 0.42). Titers of anti-SFGR- and anti-TGR-specific IgG detected in human sera ranged from 100 to 6400. The geometric mean titers were 673 for anti-TGR IgG and 635 for anti-SFGR antibodies.

**Table 1:** Seroprevalence of antibodies against TGR and SFGR in humans and small mammals from Tsiroanomandidy, Madagascar. Exact binomial confidence intervals (95% CI) are given in square brackets

Tested sera	Area	No. sampled	No. anti-TGR IgG positive (%) [95% CI]	No. anti-SFGR IgG positive (%) [95% CI]
Human	Andrefanigara	31	14 (45.2) [27.8–63.7]	9 (29) [14.9–48.2]
	Ambarivatry	31	10 (32) [17.3–51.5]	12 (39) [22.4–57.7]
	Total	62	24^a^ (39) [26.9–52]	21^a^ (34) [22.6–47.1]
*Rattus rattus* ^b^	Andrefanigara	44	2 (4.5) [0.8–16.7]	0
	Ambarivatry	40	0	0
	Total	84	2 (2.4) [0.4–9.1]	0

^a^Six individuals were seropositive for both anti-TGR and anti-SFGR IgG

^b^Sera samples from 2 *Suncus murinus* captured in Andrefanigara were also tested, both were negative

Two rat samples from the 46 small mammals collected in the Andrefanigara were positive for anti-TGR antibodies based on the ratio method (Table 1). The titers of antibodies against TGR detected in the 2 *R. rattus* were > 6400. No rodent from Ambarivatry presented with antibodies to TGR, and anti-SFGR IgG were not detected in small mammal sera from either area.

Results of rickettsial DNA detection in fleas from Tsiroanomandidy are shown in Table 2. Of the *X. cheopis* fleas tested from 19 rats and one mouse caught in Andrefanigara (*n* = 107), 24% were positive for *R. typhi* DNA and 2% for *R. felis* DNA. Fleas positive for *R. typhi* DNA were collected from eight rats, and for these rats the proportion of positive fleas ranged from 7% (*n* = 15 fleas) to 100% (four rats, *n* = 1, 6, 2, 3), with a median of 90%, indicating a very clumped distribution of infected fleas. Rats from houses and their immediate vicinity were not more likely to carry infected fleas (42%, *n* = 14) than those from outdoor lines around vegetation (40%, *n* = 5). Of the *P. irritans* fleas tested from Miandrarivo, 31% (*n* = 26) were positive for *R. felis* DNA.

**Table 2:** Detection of rickettsial DNA in fleas collected from rodents and houses from Tsiroanomandidy, Madagascar. Exact binomial confidence intervals (95% CI) are given in square brackets

Flea species	Principal host	Location	No. sampled	*Rickettsia* spp*.* positive (%) [95% CI]	*R. typhi* positive (%) [95% CI]	*R. felis* positive (%) [95% CI]
*X. cheopis* ^a^	Rodents	Andrefanigara	107	36 (33.6) [25–43.5]	26^b^ (24.3) [16.8–33.7]	2^b^ (1.9) [0.3–7.2]
*P. irritans*	Humans	Miandrarivo	26	15 (57.7) [37.2–76]	0	8^b^ (30.8) [15.1–51.9]

^a^Other fleas collected from rodents were all negative: *S. fonquerniei* from Andrefanigara (*n* = 4) and Ambarivatry (*n* = 5) and *X. cheopis* from Ambarivatry (*n* = 1)

^b^Most *Rickettsia* spp. positive samples that were not subsequently positive for *R. typhi* or *R. felis* had Ct values close to 40

**Fig. 2:**
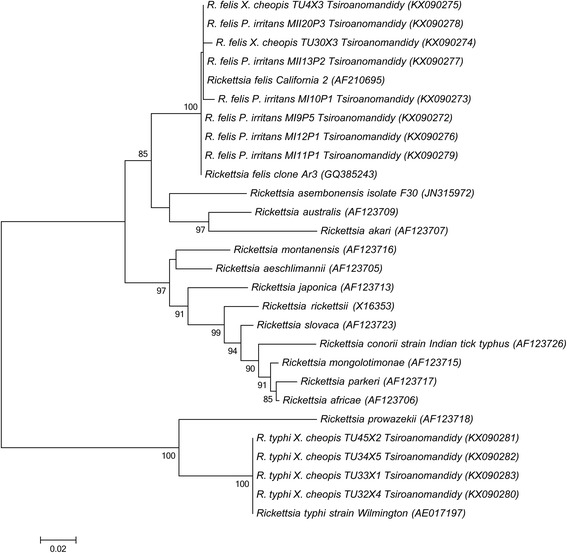
Maximum likelihood phylogeny of *Rickettsia* species detected in fleas from Tsiroanomandidy based on *ompB* gene. Phylogenetic analysis was performed with 801 bp and 795 fragment of *ompB* gene of *R. felis* and *R. typhi*, respectively, with GTR + G as the best DNA substitution model. Bootstrap values > 80% are presented. The scale bar on the bottom of the tree represents the number of substitutions per site. GenBank accession numbers are provided within brackets

*Rickettsia* spp. *ompB* sequences were obtained from 6 *P. irritans* from Miandrarivo and 6 *X. cheopis* from Andrefanigara (GenBank accession numbers KX090272–KX090283). These sequencing data were consistent with results from the species-specific qPCR assays and revealed four variants of *R. felis* and a single variant of *R. typhi* (Fig. 2): *R. felis* from 2 *X. cheopis* and 3 *P. irritans* were 99.4–100% identical to *R. felis* California 2, *R. felis* from 3 *P. irritans* were 99.9–100% identical to *R. felis* clone Ar3 and *R. typhi* from 4 *X. cheopis* were 100% identical to *R. typhi* strain Wilmington. The best DNA substitution model fitting the data was determined to be GTR + G. The maximum likelihood phylogenetic tree based on these fragments of *ompB* gene is shown in Fig. 2.

## Discussion

Rickettsial infections occur worldwide and may cause serious illness for humans. Recent studies in Madagascar focussed on tick-borne Rickettsiae [2, 3]. We reported here the first molecular evidence of *R. typhi* and *R. felis* in fleas from Madagascar. *Rickettsia typhi* was found in *X. cheopis* fleas from black rats, with rats in proximity to houses having higher flea infestations. *Rickettsia felis* was also closely associated with humans, being found predominantly in *P. irritans*. Consistent with our findings of peridomestic fleas infected with rickettsial pathogens from both groups, we found evidence within the human populations studied of previous exposure to both TGR and SFGR pathogens.

A previous study of apparently healthy participants in seven African countries that used an indirect immunofluorescent antibody assay (IFA) found seroprevalences of antibodies against *R. conorii*, a SFGR member, ranging from 0% in the Comoros to 46% in Angola, whilst seroprevalences of antibodies against *R. typhi* ranged from 0% in Angola to 11% in Mali [4]. Interestingly, some of the lowest prevalences were in the Comoros (TGR 2%; SFGR 0%), a group of islands close to Madagascar. A recent study, also using *R. conorii* as an SFGR antigen in an IFA test, and samples from pregnant women from six sites in Madagascar, found a low seroprevalence of anti-SFGR antibodies (0.5–3.1%) [2]. These results are compared with our anti-SFGR and anti-TGR IgG seroprevalences of 34 and 39%, respectively. Thus, our results from an anti-SFGR ELISA fall within the wide range of seroprevalences found in other African countries, but are much higher than the recent study in Madagascar. Interestingly, in our study we observed that females showed a lower seroprevalence for SFGR (19%) than males (44%). The low seroprevalence in the Keller et al. [2] study may therefore be related to the group sampled. Further work is needed to assess whether the prevalence of antibodies to SFGR differs between groups within the population, between sites or whether differences between studies are predominantly due to differing sensitivities of diagnostic tests. Compared with results from other African countries our TGR prevalence appears unusually high. With one of the highest incidences of plague in the world, the human population in Madagascar has a known exposure to diseases transmitted by rats and *X. cheopis* fleas, and Tsiroanomandidy district has a particularly high plague incidence [18]. Thus high exposure to *X. cheopis* fleas may explain the high exposure rate to TGR. A study in Malang, Indonesia, a city with a history of plague, reported similarly high anti-TGR IgG seroprevalences, with a significant increase from rural to suburban to urban population (28, 33 and 42%, respectively) [19]. We found no evidence of a significant difference in seroprevalence between urban and rural human populations in Tsiroanomandidy district, despite a much higher abundance of *X. cheopis* fleas in the urban site and a lack of evidence of infection in the few *X. cheopis* sampled at the rural site. Clearly, our flea data represented a single time point, whilst seroprevalence in the human population reflected exposure rates over time, and more data are needed to assess urban–rural differences in flea abundance, flea infection rates and anti-TGR IgG seroprevalence in the human population.

Anti-TGR and SFGR IgG were detected in six human sera. Thus 25% of samples that reacted to TGR antigens also reacted to SFGR antigens. This is a similar proportion to the findings reported in the Dupont et al. [7] study of apparently healthy participants (33%) [4], but much lower than the proportion of dual-positive samples reported during a study on febrile patients from Kenya (90%) [20]. Cross-reactivity between SFGR and TGR antigens had been reported [21, 22], and there had been suggestion that antibodies to *R. felis* may be a primary source of these cross-reactions [23]. However, more recently a study using the same ELISA approach as used here reported that antibodies directed against *R. felis* reacted with SFGR antigens, but not with TGR antigens [24]. Thus, our dual-positive sera may reflect previous exposure to both TGR and SFGR pathogens.

Whilst the group-specific ELISA tests are useful for an epidemiological serosurvey, the gold standard for diagnostics is IFA, followed by western blot, as this is more specific and can provide information on the most likely infecting *Rickettsia* sp*.* Apart from *R. typhi*, the TGR also includes *R. prowazekii*. As this louse-borne pathogen is most common in Africa in cooler, high altitude areas [25, 26] and our data indicated high prevalence of *R. typhi* in fleas, we believe exposure to *R. typhi* is more likely. A range of SFGR are known to occur in Africa, with most of these transmitted by ticks [27], and our SFGR results may, therefore, reflect an exposure to multiple SFGR pathogens*.* In the Dupont et al. [7] study, a subset of samples with a high titre to *R. conorii* antigens by IFA were used in a western blot against *R. conorii* and *R. africae* (another SFGR group member). The results indicated that the majority of sera reacted most strongly against *R. africae* [4]. Moreover, whereas the main ticks associated with transmission of *R. conorii*, *Rhipicephalus* spp., are found throughout Africa, the prevalence of antibodies to SFGR appeared highest in areas where *Amblyomma* ticks (commonly associated with cattle) are found. This led Dupont et al. [7] to speculate that much of the exposure to SFGR in sub-Sahelian Africa may be due to *R. africae* rather than *R. conorii*. However, since this study, several studies have found that *R. felis* may be an important cause of febrile disease in Africa [12, 20, 28]. A recent study found a high prevalence of *R. africae* in *Amblyomma* ticks collected from cattle in Madagascar but, as described above, a low seroprevalence of anti-SFGR antibodies in pregnant women using an IFA test [2]. In the same study, a further IFA against *R. africae* antigen with strong positive samples from the original test indicated titres suggestive of previous *R. africae* infection. However, reactivity to *R. felis* antigens was not assessed. It was not, therefore, currently possible to ascertain the proportion of the population exposed to *R. africae*, *R. felis* or other SFGR species and further work is needed to confirm the identity of SFGR infecting the Malagasy population.

Awareness of rickettsioses amongst clinicians in Madagascar is low. Given the high seroprevalences of antibodies to *Rickettsia* spp. in the general population of Tsiroanomandidy district, rickettsioses may be under-diagnosed as causes of undifferentiated fever, as had been reported in studies of febrile patients in Kenya, Tanzania and Senegal [12, 20, 29, 30]. These previous studies confirmed the potential of both *R. typhi* and *R. felis* to cause disease in humans. Similar studies are needed to determine the role of rickettsial agents in febrile syndromes in Madagascar.

Our results indicate that *X. cheopis* is likely to be a major vector for murine typhus in Madagascar, as has been found elsewhere [5]. *Rickettsia felis* is most commonly associated with the cat flea, *Ctenocephalides felis* [4, 31]. However, it had also been found in other flea species, including *X. cheopis* [32–34]. A study in the Democratic Republic of Congo using an unspecified *R. felis-*specific qPCR reported *R. felis* in *P. irritans* [35]. More recently, in Senegal, although the same *R. felis*-specific qPCR assay as used in this study detected rickettsial DNA in *P. irritans* fleas, further testing revealed that this was actually detection of a new *Rickettsia* sp*.*, *Rickettsia asembonensis*, which is closely related to *R. felis* but not known to cause disease in humans [15, 36]. Our *R. felis ompB* sequence from *P. irritans* in Madagascar confirmed that this flea species can carry *R. felis*. However, the epidemiological cycle of *R. felis* in Africa remained unclear, with *R. felis* detected in a number of other arthropods, including mosquitoes and bed bugs [37].

Although we detected rickettsial DNA in *X. cheopis* collected from rats, seroprevalence of rat antibodies against *Rickettsia* spp. in this site was surprisingly low, with only two *R. rattus* out of 84 positive with anti-TGR IgG. One of the two seropositive rats carried no fleas, whilst the second carried 13 *X. cheopis* fleas, one of these fleas was positive for *R. felis* by qPCR. Our seroprevalence compares with high seroprevalence detected in several other studies. Antibodies to *R. typhi* were detected in 23.7% of rodents in Thailand [38], 11% of *Rattus* captured in Jayapura, Indonesia [39] and 35.9% of peridomestic rodents in Malang, Indonesia [19]. Our low seroprevalence may be due to a lack of sensitivity of our ELISA technique for rat sera, as we did not have a positive control that showed strong OD readings with the anti-rat conjugate for optimising the test (the mouse positive controls for both TGR and SFGR worked well with the protein-A peroxidase but consistently showed low OD readings with the anti-rat conjugate. Thus, we may have underestimated the number of rats with previous exposure to TGR. Although other mammals can act as reservoirs for murine typhus [30], it seems likely that *R. rattus* do play a role in Madagascar given their known reservoir capacity [5, 39] and the high percentage of *R. typhi* positive fleas feeding on them.

## Conclusion

We found evidence here of exposure to both TGR and SFGR pathogens in the general human population, suggesting that rickettsioses should be considered as potential causes of undifferentiated fever in Madagascar. Molecular evidence demonstrates the presence of *R. typhi* and *R. felis* in fleas. We also confirm that the human flea, *P. irritans*, can be infected with *R. felis*, a *Rickettsia* species recently highlighted as an agent of febrile disease elsewhere in Africa. Further testing of human sera and flea samples from other districts is currently being conducted, and will provide more detail on the prevalence, distribution and risk of *Rickettsia* infections.

## References

[1] Renvoisé A, Raoult D (2009). L’actualité des rickettsioses. Med Mal Infect.

[2] Keller C, Krüger A, Schwarz NG, Rakotozandrindrainy R, Rakotondrainiarivelo JP, Razafindrabe T (2016). High detection rate of *Rickettsia africae* in *Amblyomma variegatum* but low prevalence of anti-rickettsial antibodies in healthy pregnant women in Madagascar. Ticks Tick Borne Dis.

[3] Ehlers J, Ganzhorn JU, Silaghi C, Krüger A, Pothmann D, Ratovonamana RY (2016). Tick (*Amblyomma chabaudi*) infestation of endemic tortoises in southwest Madagascar and investigation of tick-borne pathogens. Ticks Tick Borne Dis.

[4] Azad AF, Radulovic S, Higgins JA, Noden BH, Troyer JM (1997). Flea-borne rickettsioses: ecologic considerations. Emerg Infect Dis.

[5] Azad AF (1990). Epidemiology of murine typhus. Annu Rev Entomol.

[6] Balleydier E, Camuset G, Socolovschi C, Moiton M, Kuli B, Foucher A (2015). Murine typhus, Reunion, France, 2011–2013. Emerg Infect Dis.

[7] Dupont HT, Brouqui P, Faugere B, Raoult D (1995). Prevalence of antibodies to *Coxiella burnetti*, *Rickettsia conorii*, and *Rickettsia typhi* in seven African countries. Clin Infect Dis.

[8] Ratovonjato J, Randriambelosoa J, Robert V (2008). *Tunga penetrans* (Insecta, Siphonaptera, Tungidae) à Madagascar: une nuisance négligée. Rev Med Vet.

[9] Graf PCF, Chretien J-P, Ung L, Gaydos JC, Richards AL (2008). Prevalence of seropositivity to spotted fever group Rickettsiae and *Anaplasma phagocytophilum* in a large, demographically diverse US sample. Clin Infect Dis.

[10] Duchemin J-B. Biogéographie des puces de Madagascar. [Thèse de doctorat]: Université de Paris XII–Val de Marne; 2003.

[11] Cornel AJ, Collins FH (1996). PCR of the ribosomal DNA intergenic spacer regions as a method for identifying mosquitoes in the *Anopheles gambiae* complex. Methods Mol Biol.

[12] Socolovschi C, Mediannikov O, Sokhna C, Tall A, Diatta G, Bassene H (2010). *Rickettsia felis*-associated uneruptive fever, Senegal. Emerg Infect Dis.

[13] Henry KM, Jiang J, Rozmajzl PJ, Azad AF, Macaluso KR, Richards AL (2007). Development of quantitative real-time PCR assays to detect *Rickettsia typhi* and *Rickettsia felis*, the causative agents of murine typhus and flea-borne spotted fever. Mol Cell Probes.

[14] Roux V, Raoult D (2000). Phylogenetic analysis of members of the genus *Rickettsia* using the gene encoding the outer-membrane protein rOmpB (ompB). Int J Syst Evol Microbiol.

[15] Jiang J, Maina AN, Knobel DL, Cleaveland S, Laudisoit A, Wamburu K (2013). Molecular detection of *Rickettsia felis* and *Candidatus Rickettsia asemboensis* in fleas from human habitats, Asembo, Kenya. Vector Borne Zoonotic Dis.

[16] Tamura K, Stecher G, Peterson D, Filipski A, Kumar S (2013). MEGA6: Molecular Evolutionary Genetics Analysis version 6.0. Mol Biol Evol.

[17] R Core Team (2014). R: A language and environment for statistical computing.

[18] Andrianaivoarimanana V, Kreppel K, Elissa N, Duplantier JM, Carniel E, Rajerison M (2013). Understanding the persistence of plague foci in Madagascar. PLoS Negl Trop Dis.

[19] Richards AL, Soeatmadji DW, Widodo MA, Sardjono TW, Yanuwiadi B, Hernowati TE (1997). Seroepidemiologic evidence for murine and scrub typhus in Malang, Indonesia. Am J Trop Med Hyg.

[20] Maina AN, Knobel DL, Jiang J, Halliday J, Feikin DR, Cleaveland S (2012). *Rickettsia felis* infection in febrile patients, Western Kenya, 2007–2010. Emerg Infect Dis.

[21] Ormsbee R, Peacock M, Philip R, Casper E, Plorde J, Gabre-Kidan T (1978). Antigenic relationship between the typhus and spotted fever group of Rickettsiae. Am J Epidemiol.

[22] Hechemy KE, Raoult D, Fox J, Han Y, Elliott LB, Rawlings J (1989). Cross-reaction of immune sera from patients with rickettsial diseases. J Med Microbiol.

[23] Znazen A, Rolain J-L, Hammami N, Hammami A, Jemaa MB, Raoult D (2006). *Rickettsia felis* infection, Tunisia. Emerg Infect Dis.

[24] Maina AN, Fogarty C, Krueger L, Macaluso KR, Odhiambo A, Nguyen K (2016). Rickettsial infections among *Ctenocephalides felis* and host animals during a flea-borne rickettsioses outbreak in Orange County, California. PLoS One.

[25] Mokrani K, Fournier PE, Dalichaouche M, Tebbal S, Aouati A, Raoult D (2004). Reemerging threat of epidemic typhus in Algeria. J Clin Microbiol.

[26] Perine PL, Chandler BP, Krause DK, McCardle P, Awoke S, Habte-Gabr E (1992). A clinico-epidemiological study of epidemic typhus in Africa. Clin Infect Dis.

[27] Berrelha J, Briolant S, Muller F, Rolain J-M, Marie J-L, Pagés F (2009). *Rickettsia felis* and *Rickettsia massiliae* in Ivory Coast, Africa. Clin Microbiol Infect.

[28] Parola P (2011). *Rickettsia felis*: from a rare disease in the USA to a common cause of fever in sub-Saharan Africa. Clin Microbiol Infect.

[29] Crump JA, Morrissey AB, Nicholson WL, Massung RF, Stoddard RA, Galloway RL (2013). Etiology of severe non-malaria febrile illness in northern Tanzania: a prospective cohort study. PLoS Negl Trop Dis.

[30] Richards AL, Jiang J, Omulo S, Dare R, Abdirahman K, Ali A (2010). Human infection with *Rickettsia felis*, Kenya. Emerg Infect Dis.

[31] Reif KE, Macaluso KR (2009). Ecology of *Rickettsia felis*: a review. J Med Entomol.

[32] Eremeeva ME, Warashina WR, Sturgeon MM, Buchholz AE, Olmsted GK, Park SY (2008). *Rickettsia typhi* and *R. felis* in rat fleas (*Xenopsylla cheopis*), Oahu, Hawaii. Emerg Infect Dis.

[33] Jiang J, Soeatmadji DW, Henry KM, Ratiwayanto S, Bangs MJ, Richards AL (2006). *Rickettsia felis* in *Xenopsylla cheopis*, Java, Indonesia. Emerg Infect Dis.

[34] Christou C, Psaroulaki A, Antoniou M, Toumazos P, Ioannou I, Mazeris A (2010). *Rickettsia typhi* and *Rickettsia felis* in *Xenopsylla cheopis* and *Leptopsylla segnis* parasitizing rats in Cyprus. Am J Trop Med Hyg.

[35] Sackal C, Laudisoit A, Kosoy M, Massung R, Eremeeva ME, Karpathy SE (2008). *Bartonella* spp. and *Rickettsia felis* in fleas, Democratic Republic of Congo. Emerg Infect Dis.

[36] Maina AN, Luce-Fedrow A, Omulo S, Hang J, Chan T-C, Ade F (2016). Isolation and characterization of a new *Rickettsia* species (*Rickettsia asembonensis sp. nov*) obtained from cat fleas (*Ctenocephalides felis*). Int J Syst Evol Microbiol.

[37] Mediannikov O, Socolovschi C, Edouard S, Fenollar F, Mouffok N, Bassene H (2013). Common epidemiology of Rickettsia felis infection and malaria, Africa. Emerg Infect Dis.

[38] Chareonviriyaphap T, Leepitakrat W, Lerdthusnee K, Chao CC, Ching WM (2014). Dual exposure of *Rickettsia typhi* and *Orientia tsutsugamushi* in the field-collected *Rattus* rodents from Thailand. J Vector Ecol.

[39] Richards AL, Rahardjo E, Rusjdi AF, Kelly DJ, Dasch GA, Church CJ (2002). Evidence of *Rickettsia typhi* and the potential for murine typhus in Jayapura, Irian Jaya, Indonesia. Am J Trop Med Hyg.

